# Animal Models for Influenza Viruses: Implications for Universal Vaccine Development

**DOI:** 10.3390/pathogens3040845

**Published:** 2014-10-21

**Authors:** Irina Margine, Florian Krammer

**Affiliations:** 1 Department of Microbiology, Icahn School of Medicine at Mount Sinai, New York, NY 10029, USA; E-Mail: irina.margine@mssm.edu; 2 The Graduate School of Biomedical Sciences, Icahn School of Medicine at Mount Sinai, New York, NY 10029, USA

**Keywords:** influenza virus, heterosubtypic immunity, influenza animal models, universal influenza virus vaccine

## Abstract

Influenza virus infections are a significant cause of morbidity and mortality in the human population. Depending on the virulence of the influenza virus strain, as well as the immunological status of the infected individual, the severity of the respiratory disease may range from sub-clinical or mild symptoms to severe pneumonia that can sometimes lead to death. Vaccines remain the primary public health measure in reducing the influenza burden. Though the first influenza vaccine preparation was licensed more than 60 years ago, current research efforts seek to develop novel vaccination strategies with improved immunogenicity, effectiveness, and breadth of protection. Animal models of influenza have been essential in facilitating studies aimed at understanding viral factors that affect pathogenesis and contribute to disease or transmission. Among others, mice, ferrets, pigs, and nonhuman primates have been used to study influenza virus infection *in vivo*, as well as to do pre-clinical testing of novel vaccine approaches. Here we discuss and compare the unique advantages and limitations of each model.

## 1. Introduction

### 1.1. The Virus

The influenza virus is a widespread, highly transmissible and rapidly evolving pathogen that causes influenza—an acute respiratory disease. Influenza viruses can be classified as A, B, and C viruses, with the first two causing significant disease in humans. They belong to the *Orthomyxoviridae* family and possess a single-stranded, negative-sense RNA genome, which is spread over eight segments: PB1 (polymerase basic protein 1), PB2 (polymerase basic protein 2), PA (polymerase acidic protein), NP (nucleoprotein), HA (hemagglutinin), NA (neuraminidase), M (matrix), and NS (nonstructural protein). The eight segments code for at least ten proteins including PB1, PB2, PA, NP, HA, NA, M1, M2, NS1, and NS2 [1]. The antigenic properties of the two surface glycoproteins, the hemagglutinin (HA) and the neuraminidase (NA), are used to further sub-classify influenza A viruses strains expressing different combinations of 16 HA (group 1: H1, H2, H5, H6, H8, H9, H11, H12, H13, H16; and group 2: H3, H4, H7, H10, H14, H15) (figure 1A) and nine NA subtypes (N1-9) have been isolated so far [1];. In addition, sequences of H17N10 and H18N11 viruses have been recently detected in bats as well [2,3]. Of all, only the H1N1, H2N2, and H3N2 variants have fully adapted to circulate in humans [4,5], but others such as H5N1 [6], H9N2 [7], H7N3 [8], H7N7 [9] and more recently H6N1 [10], H7N9 [11], and H10N8 [12] viruses are known to cause sporadic infections and are considered to be of pandemic potential. Circulating influenza B viruses belong to two different lineages and are either Yamagata-like or Victoria-like.

#### Influenza

In humans, seasonal influenza may commence with respiratory symptoms that resemble the common cold—such as nasal congestion and discharge, dry cough, and sore throat. However, it tends to develop fast into a much more systemic disease characterized by a sudden onset of high fever, severe malaise, headache, myalgia, and acute anorexia [13]. In uncomplicated influenza, most of the infection is limited to the upper respiratory tract, and although cough may persist for longer periods of time, people generally recover from fever and the other systemic symptoms within 7–10 days without requiring medical attention. The pathogenesis of the virus is in part defined by tissue tropism, which is determined by the distribution of virus specific receptors (sialo-saccharides present on host cell surfaces), throughout the respiratory and gastrointestinal tract. Influenza viruses can bind two types of receptors: sialic acid—α2,6-Gal-terminated (Sia2,6) and sialic acid—α2,3-Gal-terminated (Sia2,3) saccharides. Human influenza A viruses preferentially recognize the former type while the avian strains, for example, have a predominant specificity for the latter receptors [14]. These receptor specificities correlate with the preponderance of sialyl-conjugates in the different species these viruses infect, suggesting that receptor recognition is a determinant for influenza virus host tropism. By using sialic acid specific lectins, Baum and Paulson showed that Sia2,6 is predominantly expressed in human trachea epithelium, which explains the characteristic upper respiratory tract patterns of infection for seasonal strains in humans [15].

In contrast to the seasonal-like disease, infections with avian strains such as the highly pathogenic H5N1, for example, can progress to lower respiratory disease and lead to acute viral pneumonia, aggravated by acute respiratory distress syndrome (ARDS), shock, and organ failure in humans [16]. This reflects the differential viral tropism of these strains, which seem to predominantly recognize cells of the lower respiratory tract. Van Riel and colleagues (2003) found that H5N1 strains preferentially bound to cells and macrophages in the pulmonary tissue [17]. Another pathogenesis component of some highly pathogenic H5N1 strains is a prolonged cytokine storm triggered upon infection, particularly tumor necrosis factor (TNF)-alpha and interferon (IFN) beta, which were found to be elevated in deadly cases compared to patients who survived the infection [18]. Pathological examinations of tissues from fatal human cases revealed diffuse alveolar damage, interstitial fibrosis, bronchiolitis, hemorrhages, and abundant presence of macrophages in the lung [19].

### 1.2. Clinical Relevance and Control Measures for Influenza Virus Infections

The societal impact of these infections cannot be overestimated—they killed millions during the documented pandemics in the past [20], while epidemics resulting in about 250,000–500,000 deaths worldwide a year remain a serious public health burden [21]. Moreover, when considering direct medical and hospitalization expenses, as well as work absenteeism, influenza was estimated to be associated with an annual cost of $12 to 14 billion in the US alone [22]. 

The next pandemic influenza is impossible to predict, but the annual epidemics have been extensively documented to recur in a highly predictable seasonal pattern—in the Northern hemisphere viruses usually circulate between November and March every year, while in the Southern latitudes influenza viruses tend to cause infections from May to September. Tropical regions, where the number of infections can be observed throughout the year, make an exception [23]. Various factors have been hypothesized to play a role in influenza’s seasonal contagiousness [24,25], but the exact mechanism is not fully understood, and for this reason the study of transmission and ways to prevent it remain very relevant. 

Strategies to control and contain seasonal influenza epidemics include public-health measures to restrict the outbreaks, antiviral medication, and vaccination. Two classes of drugs are in use for prophylaxis and treatment of influenza: small molecule blockers of the M2 ion channel (amantadine and rimantadine) [26] and NA inhibitors (oseltamivir, zanamivir and peramivir—reviewed in [27]). Most of the influenza A and B viruses are resistant to the first class of drugs [28], leaving prophylaxis to rely mostly on neuraminidase inhibitors. Though to a lesser extent, the use of these compounds also associates with occurrence of resistant strains [29]. Consequently, vaccination remains the most efficient and cost effective countermeasure against the virus [30]. 

Seasonal influenza vaccines have been available since the 1940s and usually have a good efficacy in preventing symptomatic influenza infection [31]. At present, three types of influenza vaccines are licensed by the FDA—an inactivated influenza vaccine, a live-attenuated influenza vaccine (LAIV) and a recombinant protein vaccine [32,33].

All formulations contain antigens of currently circulating influenza strains—an H1N1, H3N2, and influenza B virus components belonging to both lineages (quadrivalent vaccines). They are designed to primarily elicit an immune response to the major antigen of the influenza virus—the HA protein. This protein is expressed as a homotrimer on the surface of the influenza virus and mediates receptor recognition as well as subsequent fusion of the viral membrane in the endosome [1]. These two functions can be structurally mapped to two distinct domains, the “globular head” and the “stalk” domain, respectively. When there is a good match between the vaccine antigens and the circulating strains, seasonal vaccines have a high efficacy in preventing disease (up to 90% in healthy adults [34,35]). However, due to the constant antigenic evolution of these viruses, formulations have to be changed every year by following a prediction process that relies on surveillance data [31]. Thus, the accuracy of these predictions determines the vaccine efficacy. Although in most years the process is successful, drifted variants and mismatches with the predicted vaccine strains have occurred [36,37]. Furthermore, in the case of newly emerging strains, there is a gap between the outbreak onset and the availability of subtype-specific vaccine supplies, as highlighted by the swine-origin H1N1 pandemic in 2009 [38].

One way to enhance our preparedness for influenza epidemics and pandemics would be to develop a universal influenza virus vaccine. Many research efforts have focused on this [39], but one of the most promising approaches targets the conserved stalk domain of the HA protein on the surface of the virus. Considering the unchanging nature of this domain, these vaccines could provide broad protection against divergent strains, and as such would overcome the limitations of yearly vaccination which mostly elicits responses against the highly variable head domain of the HA protein (for an extensive discussion see [40]).

Aside from the ever-changing and unpredictable nature of the virus, a major challenge in influenza research is the selection of an appropriate animal model that accurately reflects the disease and, in the case of vaccine studies—the protective immune response to influenza infection—in humans. This review discusses, contrasts, and compares the advantages and limitations of the most commonly used laboratory models that have been developed and used so far for influenza virus research and vaccine development.

### 1.3. Animal Models Used in Influenza Virus Research

The earliest observations of influenza being caused by an infectious agent were made in the 1930s [41]. Ever since, researchers have been developing methods to study the virus in a controlled laboratory setting. The first success was the infection of ferrets by intranasal instillation of throat washes obtained from influenza patients [41]. Since then, several mammalian models have been extensively characterized for use in influenza research, and we now have extensive experience with combining viral strains, conditions, and the optimal models depending on the specific research questions being addressed, as discussed below. To study a specific research question and choose the optimal model system, a comprehensive understanding of the pathogenesis of the virus in humans and animals is required.

## 2. Pathogenesis of the Influenza Virus

### 2.1. Ferrets

Ferrets were the first species to be successfully infected with human influenza isolates in the early 1930s [41,42], and have since been considered the ideal small animal model for influenza research. One of the factors that contributes most to the validity of this model is the susceptibility of the animals to a wide range of human isolates without prior adaptation, including seasonal H1N1 [43,44,45], pandemic 2009 H1N1 [45,46,47], H2N2 [48,49], H3N2 [45,47,50], H5N1 [47,51], H7 subtypes [52], H9N2 [53,54,55], as well as influenza B viruses [45]. This allows for efficient and accurate testing of primary field isolates without worrying for adaptation-related antigenic or phenotypic changes. Furthermore, upon infection with seasonal influenza, the animals exhibit upper respiratory infection patterns and clinical symptoms similar to the ones in humans. An early study of Smith, Andrewes, and Laidlaw described in 1933 that upon inoculation with nasal washes collected from influenza patients, ferrets emulated most clinical symptoms generally associated with human influenza—including fever, nasal discharge, lethargy, and weakness (Table 1) [41]. It later became evident that these common features mirror a similar lung physiology that ferrets and humans share; this was in agreement with a similar distribution of sialic acid containing receptors in the respiratory tract of both species [56]. The presence and severity of symptoms, however, varies greatly depending on the viral strain and the route of administration—and can range from no apparent clinical signs to severe anorexia and weight loss, and death [57]. The outcome is also age dependent with young, newly weaned ferrets showing less symptoms than adult ones [58]. The most extreme outcomes are usually observed upon infection with highly pathogenic avian influenza A viruses, which can spread to extrapulmonary organs. In these cases other symptoms such as diarrhea and neurological damage may occur [59].

Histopathologically, infection with seasonal influenza A viruses induces bronchiolitis and interstitial pneumonia [60], while highly pathogenic viruses were found to provoke extensive bronchiolar inflammation, necrosis of bronchial epithelium, and suppurative exudates in the bronchiolar lumen [61]. The pronounced differences in pathology between seasonal and pandemic viruses correlate well with the results of histochemical studies that described the predisposition of the former viruses to attach to cells in the ferret upper respiratory tract, while H5N1 isolates attached preferentially to alveoli and bronchioles in the lower respiratory tract [56,62]. These observations provide an explanation for the different disease outcomes, and also highlight the similarities ferrets share with humans in this regard. The effectiveness of this model is also underlined by the ability of infected ferrets to transmit human non-adapted seasonal and pandemic viruses to naïve animals through either direct contact or respiratory droplets [41,63,64]. In fact, currently the ferret is the only mammalian model that is equally suitable for the study of both pathogenesis and transmission of influenza viruses. Their use can be limited, however, due to lack of ferret-specific immunological reagents and incomplete genome sequencing of this species, as discussed in the vaccine section. Other potential drawbacks of this system include high costs, size and husbandry requirements for them, making the use of large group numbers of animals prohibitively expensive. Use of fewer animals per group can lower the significance of the statistical analysis that can be performed, sometimes limiting the conclusions drawn.

### 2.2. Mice

Mice are extensively used for influenza research. Even though wild mice are not natural hosts for influenza viruses, laboratory strains can be infected with certain influenza viruses. The viruses generally require adaptation to be able to replicate and achieve virulence, but the degree of susceptibility of these animals to influenza also depends on the mouse strain (for an extensive review see [13]), and it was shown to be significantly increased by deletions in the *Mx1* antiviral gene [65,66]. Adaptation of the virus usually implies repeatedly passaging it *in vivo* in mouse lungs, a process that results in amino acid changes which improve receptor binding and allow for replication, and increase the virulence of the isolates [67,68,69]. The downside of this process is that, though infectious and virulent, the adapted virus may be very different from the initial strain—antigenically, phenotypically, or both. There are several influenza virus types, however, such as the highly pathogenic ones such as the pandemic H1N1 (1918 [70] and 2009 [64] strains), H5N1 [71,72], and several H7 strains [9,61,73], which cause disease in mice without requiring prior adaptation. These differences correlate well with the tissue distribution of influenza receptors in the mouse respiratory tract—mice were found to predominantly express Sia 2,3 type receptors throughout the upper and lower airways tissues [74]. 

Clinical signs usually develop 2–3 days after infection and vary considerably, depending on both mouse and virus strains, as well as the challenge dose. Symptoms include lethargy, anorexia and loss of bodyweight, huddling, ruffled fur, and death. Other human disease-like symptoms such as coughing, nasal discharge and fever have not been observed (Table 1). On the contrary, mice develop hypothermia upon infection, which is a common response in small animals due to their inability to control their metabolic rates under infection and stress conditions [75]. As discussed below, weight loss is the most convenient quantitative readout in vaccine effectiveness experiments.

**Table 1:** Summary of signs of disease and pathology present in the different models (depending on challenge virus strain).

Clinical Signs	Animal Model			
	Ferret	Mouse	Pig	Nonhuman Primates
**Nasal discharge**	YES	NOT OBSERVED	YES	YES
**Coughing/sneezing**	YES (sneezing)	NOT OBSERVED	OCCASIONALLY - intensity varies with the strain -	YES - only upon infection with highly pathogenic viruses -
**Malaise**	YES	YES	NOT OBSERVED	YES
**Fever**	YES	NO - develop hypothermia -	OCCASIONALLY - intensity varies with the strain -	OCCASIONALLY - only upon infection with highly pathogenic viruses -
**Anorexia** - **weight loss** -	YES - more predominant for highly pathogenic viruses -	YES	MINOR	MINOR
**Neurological complications**	OCCASIONALLY - high path avian viruses -	OCCASIONALLY - high path avian viruses -	NOT OBSERVED	NOT OBSERVED
**Hypercytokenia** - **cytokine storm** -	YES - in some cases of highly virulent strains -	YES - upon infection with highly pathogenic viruses -	LIMITED	YES

Despite these drawbacks, mice remain the most widely used animal model for influenza research, as they have advantages over other species—including small size, low cost, availability of transgenic strains with targeted gene disruptions [76] (which allow for the study of very detailed processes in host response), as well as broad accessibility of research immunological reagents [77].

### 2.3. Pigs

Influenza viruses of both avian and human origin can naturally infect pigs, which prompted pigs to be proposed as intermediate hosts for influenza viruses [78]. This correlates with data from immunostaining experiments with linkage-specific lectins that showed that both α2-3 and α2-6 linkages are expressed on epithelial cells of pig trachea [79]. Their ability to be infected by most influenza subtypes makes them an attractive model, and pigs have gained significant interest since the 2009 swine-origin H1N1 pandemic. Generally, viruses replicate in the epithelium of the entire respiratory tract, but do not disseminate to extrapulmonary organs. Signs of illness include fever, loss of appetite, labored breathing, and coughing, but their occurrence and intensity vary depending on the viral strain (Table 1) [80]. Histologic lesions may occur as tracheobronchitis and bronchointerstitial pneumonia [81], infiltration of alveolar septa by large macrophages, hyperplasia of type II pneumocytes, and free necrotic cells in the alveolar lumen [82]. Interestingly, pigs have a very low susceptibility and are asymptomatic to infection with H5N1 influenza [81].

Most of the limitations of the model are of a practical nature, such as the size of the animals and problematic husbandry requirements, and pigs are mainly used in studies aiming at developing novel vaccines for swine influenza strains [83].

### 2.4. Nonhuman Primates

Because they are closely related to humans, nonhuman primates (including Rhesus macaques, Pig-tailed macaques, and Cynomolgus macaques, Squirrel monkeys, African green monkeys) are in theory good models for human disease. They can be naturally infected by influenza viruses [84], and have been successfully infected in the laboratory with a number of unadapted human influenza isolates including seasonal H1N1 [85], H3N2 [86], 2009 pandemic H1N1 [87], 1918 pandemic H1N1 [88], high pathogenic H5N1 [88] and H7N9 viruses [89]. However, ethical issues regarding their use, the prohibitive costs, complicated husbandry requirements, and the need for extremely experienced personnel make this the least accessible model for influenza research. Furthermore, even though the viruses replicate well in the respiratory tract, animals do not generally develop any symptoms of disease upon experimental inoculation with seasonal viruses [90]. Highly pathogenic strains do induce clinical signs that are reminiscent of severe disease in humans—from fever, cough, lethargy, followed by ARDS and bronchointerstitial pneumonia [17], to even more severe peribronchiolar alveolitis, edema, and hemorrhage when exposed to a reconstructed 1918 pandemic virus [91]. The main advantage of the model is that, in vaccine and therapeutic studies, it allows for the analysis of immune reactions most closely related to the ones mounted by humans.

### 2.5. Other Animal Models

In addition to the models discussed above in detail there are several other animal species that are occasionally used for influenza vaccine studies. One of the most widely used other animal model is the guinea pig [92]. Guinea pigs are ideal animals for transmission studies since influenza viruses replicate in their upper respiratory tract at high titers [24,25,92,93,94]. Another advantage of the model is that—in comparison to ferrets—guinea pigs are very docile and relatively small, allowing for a higher number of animals per group. Additionally, influenza B viruses have been shown to transmit well in guinea pigs making this animal model the only available model for influenza B virus transmission [95]. However, guinea pigs do not develop clinical signs upon infection making them a less ideal model to test vaccine efficacy [92]. Nevertheless this animal model remains a useful tool to investigate how vaccines impact on transmission [96,97].

In addition to the guinea pig model other small rodents like hamsters [98,99,100,101,102,103,104,105] and cotton rats [106,107,108,109,110] are used occasionally for influenza virus vaccine research. Finally, influenza host species of agricultural relevance like chickens and other avian species have been used to test the respective veterinarian influenza vaccines [111,112,113,114]. Equine and canine influenza vaccines have also been tested in horses [98,115,116] and dogs [117], respectively. However, these models are not considered as standard models for testing human influenza virus vaccines.

## 3. Evaluation of Broadly Protective Influenza Vaccines in Animal Models

In an ideal setting, the effectiveness of novel influenza vaccines would be assessed in clinical trials. However, considering the low attack rate of influenza viruses (annually estimated at 5%–10% [21]), large numbers of subjects are required, which implies very high costs. Moreover, in the case of pandemic vaccines, it is not feasible to assess effectiveness in humans—since they are by definition considered to be immunologically naïve to these viruses, vaccinees could be at serious risk when exposed to experimental infections. For these particular studies, human subjects can be used to analyze the immunogenicity of the novel antigens, but their protective efficacy is solely assessed in animal models. These practical reasons underline the importance of preclinical studies in animals when it comes to deciding what are the most promising influenza virus vaccine candidates. The models described in the previous sections are all valuable tools for these studies, but their advantages and limitations have to be weighed in to decide what is the optimal one for each study. Table 2 summarizes some of the considerations one should keep in mind depending on the stage in antigen development (Box 1). Practical aspects, quality of the immune response they mount, clinical readouts, and correlates of protection should also be considered. Depending on the particularity of the study, other factors could also be relevant—for example the tissue tropism and pattern of receptor distribution are important when it comes to selecting an animal model to study live attenuated vaccines, since the vaccine strains require binding to appropriate receptors in the upper respiratory tract to allow for effective infectivity and subsequent immunogenicity.

Typically, the immunogenicity and protection activity of novel vaccine antigens are tested in preclinical studies in mice, ferrets, and nonhuman primates (in the case of pandemic vaccines), and only the ones that make it through this pipeline are considered for clinical studies in humans (Box). In addition, other species like rabbits can be used for toxicology testing of these influenza virus vaccines. These processes have been well and extensively reviewed for development of seasonal and pandemic influenza vaccines [60,118,119], and the following discussion mostly addresses the use of animal models for testing and developing vaccines targeting the conserved stalk domain of the HA.

**Table 2:** Advantages and disadvantages of the animal models in the use of vaccine research.

Species	Advantages	Disadvantages
**Mice**	√ Small size √ Low cost (animals, housing) √ Homogeneous responses - inbred, pathogen free √ Availability of molecular biology/immunology reagents √ Pathology of viral pneumonia caused by highly pathogenic viruses (1918 H1N1, HPAI H5N1) similar to humans	× Seasonal influenza virus strains need adaptation in order to achieve efficient replication and virulence × Respiratory tract anatomy and receptor distribution different from humans × Not suitable for study of live-attenuated vectored vaccinesNot suitable for transmission experiments
**Ferret**	√ Respiratory tract anatomy and receptor distribution similar to humans √ Human-like clinical signs and pathology of disease √ Human and avian influenza virus isolates replicate without prior adaptation √ Suitable for transmission experiments	× Limited availability of molecular biology/immunology reagents × Host response variability—genetically outbred × Need to confirm seronegativity to influenza × Systemic disease different than in humans × Genome not annotated × Practical considerations—use of high number of animals per group very expensive
**Pig**	√ Human and avian influenza virus isolates replicate without prior adaptation √ Availability of molecular biology/immunology reagents	× Host response variability—genetically outbred × Need to confirm seronegativity to influenza (maternal antibodies might be problematic) × Seem to mount an abnormal response in certain heterologous challenges, which has not been observed in humans or other species × Practical issues—big size, husbandry requirements
**NHP**	√ Respiratory tract anatomy and receptor distribution similar to humans √ High similarity to the human immune system √ Susceptible to non-adapted human strains √ Broad availability of molecular biology/immunology reagents	× Lack of clinical signs upon infection with seasonal strains × Host response variability—genetically outbred × Need to confirm seronegativity to influenza × Ethical concerns × Prohibitively expensive × Very experienced personnel and highly specific facilities needed × Variable degree of permissiveness for influenza virus infection and clinical signs

Box 1 **Influenza vaccine development in animal models.**

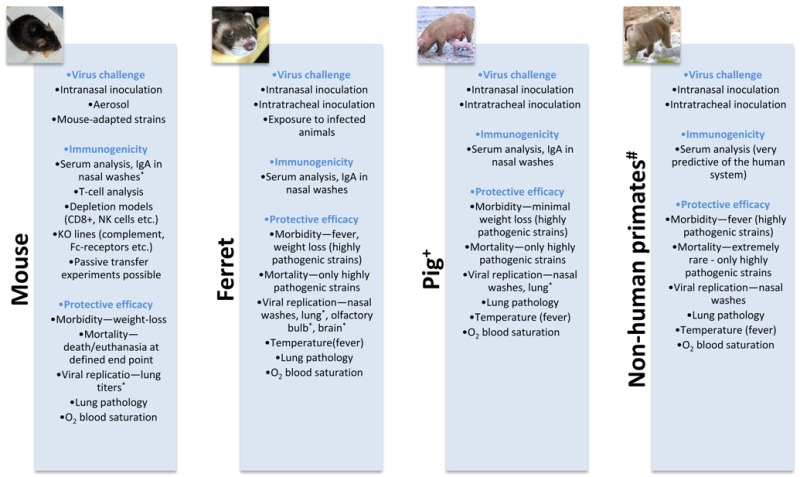

^+^ Generally used for veterinary vaccines development; # Not commonly used for influenza vaccine development^; * ^The procedure requires sacrificing the animal.

### 3.1. Mice

The first screenings of vaccine efficacy and safety are typically performed in the mouse model. This is largely due to practical considerations, which allow for effective testing of multiple vaccination schemes and conditions including antigen dose, choice of adjuvant, administration route, and large numbers of challenge virus strains. Influenza virus infections are not completely recapitulated in mice, but these animals are ideal for the study of vaccine efficacy since some of the clinical signs they experience—such as weight loss and ruffled fur—can be used to conveniently monitor infection. The utility of this model in vaccine studies is also reflected in the availability of reagents necessary for an in depth investigation of the immune responses elicited by vaccination. These include multiple antibody isotyping methods, purified cytokines, and reagents to quantify them, and oligomers of major histocompatibility complex (MHC)-peptide complexes for the quantification of virus-specific CD4+ and CD8+ T cells [120].

Furthermore, due to their inbred nature, mice tend to mount very reproducible responses. In fact the outcome of vaccine experiments can be influenced by the mouse strain used, since BALB/c mice tend to mount Th2 responses, while Th1-type immunity seems to be prevalent in C57BL/6 mice [121]. More recently, the DBA.2 mouse strain was shown to mount a humoral immune response to influenza that is qualitatively similar to that of C57BL/6 mice. The authors suggested that this strain, which appears to be more susceptible to non-adapted influenza strains than the other two, might be a suitable system for influenza vaccination studies [122]. The ability of mice to mount cross-reactive and broadly neutralizing stalk-directed humoral responses became apparent more than two decades ago [123], and ever since these processes have been extensively studied using the murine system [124,125,126]. As an indication of the utility of this model for studying cross-immunity and B memory recall, Krammer *et al.* showed that the boost in stalk antibodies elicited in humans upon pandemic 2009 H1N1 virus infection [127] could be recapitulated in mice [128]. Similarly to humans [127,129], a natural virus infection elicits limited titers of stalk-reactive antibodies, but still seems to be the most efficient way to induce these responses in mice [128,130]. On the contrary, the vast majority of the responses elicited by the currently approved inactivated vaccines are directed towards the highly variable globular head of the hemagglutinin, which contains the five antigenic sites. This domain is immunodominant over the stalk in both humans and mice [40].

Several rational vaccine strategies have been employed to enhance immunogenicity of the HA stalk domain and have been tested in mice. Steel *et al.* (2010) unmasked the region by creating “headless” HA constructs lacking the highly immunogenic globular head. Vaccination of mice with HIV gag virus-like particles containing the headless HA immunogen elicited antibodies that cross-reacted with multiple HA subtypes and protected mice from mortality in a lethal influenza challenge [131]. Though very encouraging, the responses mounted by this vaccine were limited both in breadth and in ability to protect against morbidity and weight loss. Others have tried to focus the immune response towards the stalk domain by modifying vaccination schemes and combining different prime and boost antigens. For example, DNA priming followed by protein or seasonal vaccine boost elicited good titers of cross-protective stalk antibodies [126,132]. Another approach involving vaccination with self-assembling ferritin nanoparticles exposing 8 HA protein trimers on the surface was also reported to successfully elicit such broadly neutralizing responses [133]. Even though the span of the responses elicited by these approaches was mostly limited to one HA subtype, the results were promising and furthered our understanding in how HA stalk antibodies can contribute to protection.

All the studies discussed above described strong correlations between stalk antibody induction and *in vivo* protection. This was an important first step in the field, since traditionally the only HA-specific responses believed to contribute to viral neutralization were the ones targeting the globular head domain. However, the first proof of principle experiment demonstrating that a stalk specific humoral response is sufficient to confer protection against viral challenge *in vivo* was only later performed in the mouse model. The study employed chimeric HA (cHA) constructs that expressed different exotic head domains—usually of avian origin—atop H1 (group 1) or H3 (group 2) stalk domains. It was hypothesized that sequential vaccination with constructs expressing the same stalk but divergent heads (that do not cross-react to each other) would recall memory B cells recognizing conserved epitopes in the stalk domain, while only eliciting primary responses to each different globular head. Indeed, the vaccination induced robust titers of stalk antibodies, which were sufficient to confer complete protection against both morbidity and mortality associated with a variety of heterologous and heterosubtypic virus challenges [134,135,136]. These results were later confirmed in the ferret model, but the findings in mice were pivotal for the understanding of the mechanism this vaccine works through. For example, the small size of the animals allowed the researchers to delineate the contributions of the humoral *versus* the cellular immune response by performing passive transfer experiments, with group sizes sufficiently large to permit for good statistical analysis. Further, the availability of a monoclonal antibody targeting CD8+ T cells for death, and depletion *in vivo*, provided a way to demonstrate that these cells did not have a significant role in the observed protection [135].

HA stalk directed antibodies were initially believed to inhibit virus replication solely through blocking the fusion step in the replication cycle [137]. However, recent observations suggest that alternative mechanisms such as antibody-dependent cell-mediated toxicity (ADCC), inhibition of HA maturation and viral egress, and complement activation may play a role in the protection elicited by these immunoglobulins (discussed in [40]). The availability of transgenic lineages of mice is critical in unraveling these mechanisms. For example, the ability of stalk-directed antibodies to induce ADCC was demonstrated by employing a mouse model in which animals express the full array of human Fcγ receptors (FcγRs) in a genetic background lacking all mouse Fc receptors [76]. The study clearly showed that stalk antibodies required interactions of their Fc region to FcγRs for optimum virus neutralization *in vivo*, whereas the anti-HA head mAbs did not. Aside from their basic scientific aspect, these findings could also help in the development of antibody-mediated therapies. Other broadly reactive stalk antibodies were found to trigger complement mediated lysis in infected cells [138]. In the future it would be interesting to use mouse strains that are deficient in complement pathway components (C3^−/−^) [139] to study the role of this mechanism in broad antibody neutralization.

Overall, the mouse model is instrumental in identifying promising vaccines and elucidating the immunological mechanisms that mediate protection. However, as discussed below, its predictive value for successful vaccination in humans should be taken with a grain of salt, and vaccine candidates should be tested at least in a second model before proceeding to clinical trials.

### 3.2. Ferrets

Ferrets mount a potent immune response to influenza antigens, which became evident soon after the first experimental infections with influenza viruses. In an early immunological and serological study, Smith, Andrewes, and Laidlaw (1933) observed that serum collected from convalescent ferrets had virus neutralizing activity *in vitro* [41]*.* Two years later, Francis and Magill noted that ferrets that recovered from an influenza infection were resistant against reinfection with the virus 4 months later [140]. Through passive transfer experiments of serum from either naïve or convalescent animals, the authors demonstrated that the protection was mediated by neutralizing antibodies. The antigenic variations of these viruses, as well as the limited breadth of protection of the humoral responses raised against them became readily apparent, since serum from animals previously infected with a swine virus, for example, was not able to neutralize human influenza strains effectively [141]. Ever since, the ferret model has been perfected and extensively used for development of influenza vaccines.

Since ferrets experience disease symptoms that are so similar to the human disease, the assumption is that their immune responses should also be quite similar, which prompts them as an appropriate model for the investigation of vaccine effectiveness. The in depth analysis of their immunity has been hampered, however, by the limited availability of species-specific reagents for immunological assays, and the lack of detailed genome mapping. Although this remains an issue, recent developments suggest it will be resolved in the near future. For example, a recent functional genomic study furthered our understanding on pro-inflammatory cytokine regulation upon infection with an H3N2, as well as an H5N1 virus [142]. The investigators used a canine microarray to analyze the differential gene expression in response to low- *vs.* high-pathogenicity viruses, which revealed up-regulated and earlier interferon (IFN) responses in the course of the latter infection. Another study cloned and expressed the full-length ferret IFN-γ protein, which they then used to raise specific mAbs. The antibodies work in immunoblotting, enzyme-linked immunosorbent assay (ELISA) and enzyme-linked immunospot (ELISPOT) assays, and were instrumental in confirming up-regulation of IFN-γ in serum samples collected from influenza-infected animals [143]. Since IFN-γ plays a key role in the regulation of Th1-type immune response, these observations suggest that this model has a tendency towards this type of immunity. Another possibility is using reagents that have already been developed for other species. This is what Nakata and colleagues considered when analyzing cDNA sequence homology of inflammatory cytokines of ferrets and other species. Their analysis revealed high levels of conservation between IFN-γ, (IL)-1β, IL-6, IL-8, and tumor necrosis factor (TNF)-α of ferrets, dogs, and cats [144]. Furthermore, in an attempt to start dissecting the cell-mediated immunity that ferrets mount, Rutigliano *et al.* (2008) screened a collection of available monoclonal antibodies for cross-species reactivity. The authors found a mouse mAb that efficiently recognized ferret CD8+ T cells, demonstrating at the same time a significant enrichment of these cells in lungs of pneumonic ferrets—in line with what typically happens in the human lung under similar conditions [145]. The outbred nature of these animals should also be taken into consideration, since, by reflecting into the heterogeneity of the alleles they carry, it increases the weight and confidence of the responses mounted against antigens. On the downside, each ferret used in vaccinology studies should be confirmed to be entirely naïve to influenza infection, as pre-existing immunity can dramatically skew the results of these studies.

Despite the need to clarify some aspects of their immunobiology, it is clear that ferrets possess the cornerstone mechanisms for broadly protective vaccines—immunological memory and ability to mount cross-protective immunity [146], and have been used in a variety of vaccine efficacy studies (well reviewed in [118]). Several proofs of principle experiments have revealed that these animals are able to mount cross-reactive and broadly neutralizing responses directed against the stalk domain of the HA protein. In one of the earlier studies, naïve animals that were primed with plasmid DNA encoding an H1N1 influenza HA and then boosted with either seasonal vaccine or a rAd5-based HA vaccine experienced an induction of broadly neutralizing HA stalk antibodies [126]. When considering the feasibility of a stalk-based universal vaccination strategy in humans, an important aspect remains the fact that the general population has immunological experience with influenza viruses. One hypothesis of original antigenic sin suggested that the imprint established by an individual’s first influenza infection governs all antibody responses thereafter, essentially suggesting that the immune responses against new viruses or antigens are impaired. This was recently refuted by a study that showed that immunogenicity of a pandemic 2009 H1N1 vaccine was not decreased in humans and ferrets who had previous experience to seasonal H1N1 viruses [147].

To clarify the way in which the presence of pre-existent immunity influences the elicitation and boosting of such broadly protective antibodies, Wei and colleagues performed a similar experiment in animals that were influenza-immune (by pre-infection with a sublethal dose of influenza virus or by vaccination with an adenovirus-vectored vaccine). Their study suggested that previous experience to influenza did not encumber the elicitation of stalk-directed neutralizing antibodies, and that the immunogenicity of the stalk-domain could be enhanced further under certain conditions [132,148]. Though they observed a correlation with elevated IgG and IgA antibody titers, the degree of protection obtained in these proof of concept studies were quite modest—with only marginally decreased virus titers in the upper respiratory ways. Subsequent approaches proved that this was not because of a limitation of the model. When using the cHA approach they had previously tested in mice, Krammer and colleagues observed an up to 2-log decrease in viral titers in the nasal mucosa 6 days after infection in cHA vaccinated animals, which experienced a robust boost in HA stalk antibody titers [149]. The decrease in viral loads was even more pronounced in internal organs such as the olfactory bulb and nasal turbinates, but the assessment of these requires euthanasia of the animals. This is one difference of this model compared to mice—measuring virus titer reduction remains one of the most common read-outs for efficacy assessment in this model, since pronounced weight loss can only be achieved with highly pathogenic viruses, and other signs of disease can only be measured qualitatively. Other candidates that showed potential in mice were also evaluated successfully in ferrets [133,150]. As an indication of the predictive validity of the cross-reactive responses they mount—an H5N1 vaccine that provided full heterologous protection in the ferret model was found to induce antibody responses in humans to a level compliant with the criteria of the Committee for Proprietary Medicinal Products [151]. Vaccination with the currently approved influenza vaccines induces good protection in ferrets when well matched antigenically with the challenge virus [152], but the breadth is similarly limited with the one observed in humans [148]. Since they share a similar receptor distribution with humans, the ferret is also a good model for testing novel live attenuated approaches for pandemic vaccines, and some of these studies described good induction of protective heterologous immunity [52,153,154,155]. However, in other cases, ferrets were not very predictive of the replication competence and immunogenicity of live attenuated pandemic influenza virus vaccines in humans [156,157,158] and non-human primates (African green monkeys) were a better match [159]. Importantly, ferrets can also be used for testing the vaccination-mediated prevention of transmission (for more information please refer to [13,120]). 

### 3.3. Pigs

Traditionally, pigs have mostly been used for the development of swine influenza virus vaccines, for which several novel approaches are under investigation [160,161,162]. Similarly to humans [163], mice [128] and ferrets [164,165], seasonal H1N1 vaccination (inactivated or live-attenuated) induced minimal cross-reactive humoral responses, and showed marginal efficacy against a pandemic H1N1 challenge [166]. In fact, the use of inactivated seasonal vaccines is a common practice in pigs and can effectively protect against homologous viruses [167]. However, their ability to protect against heterologous challenge viruses has been erratic [167,168,169], and several studies suggested an association between mismatched inactivated vaccines and vaccine-associated enhanced respiratory disease (VAERD) [167,170,171]. For example, in a heterologous vaccination-challenge experiment in this animal model, H1N2 vaccinated pigs developed severe clinical disease, enhanced lung consolidation and potentiated microscopic lesions compared to non-vaccinated controls, upon pH1N1 infection [171]. It is of note that the vaccine did not prevent infection, and neutralizing antibodies in serum did not cross-react with the challenge virus. Though the validity of the association is clear, the exact mechanism remains to be elucidated. Also, it is highly relevant to observe that the VAERD effect appears to be specifically connected to some—but not all [172,173,174]—whole virus inactivated vaccine strains, and it is not triggered in heterologous challenge experiments following immunization with a live-attenuated influenza- [175], a non-replicating adenovirus five vector-based- [176] or a split pandemic H5N1 vaccine [177].

Recently, Khurana and colleagues have suggested that the VAERD phenomenon is mediated by anti-HA stalk antibodies [178]. After vaccination with a whole inactivated H1N2 virus vaccine, and subsequent challenge with a pandemic 2009 H1N1 strain, the animals developed enhanced disease. Upon analysis of the serum, the investigators concluded that the effect was triggered by a response to an epitope in the stalk region, and that their findings should be considered when evaluating universal influenza vaccine candidates that aim to target the conserved HA stem. This conclusion, however, is merely based on correlational observations. First and foremost, as unraveled by a phage-display assay they performed, the measured HA2 response is predominantly focused around the fusion peptide region of the HA protein, which does not mirror the polyclonal responses and stalk-specific epitopes that have been described so far for humans or mice [40]. Perhaps even more relevant is the fact that, if this disease enhancement mechanism is indeed mediated by HA stalk responses, it should have been prevalent and obvious during the 2009 pandemic. At that time, most of the adult population had immunological experience with seasonal H1 strains and it was shown that in these people, exposure to the pandemic H1 virus through either infection [127] or vaccination [179] elicited high stalk mediated neutralizing humoral responses. The possibility in humans that seasonal H1N1 immunological experience could be associated with increased risk upon pH1N1 infection, was mentioned by Skowronski and colleagues in a Canadian vaccine cohort [180,181]. Though an association was statistically apparent between preexisting immunity and hospitalization upon pH1N1 infection, the authors could not rule out occurrence of bias or confounding factors [180,181]. Importantly, similar analyses in other parts of the world did not report any negative association or correlation [182,183,184], but instead reported modest positive protection mediated by pre-existing H1N1 immunity. Last but not least, a similar VAERD phenotype has been observed in the past upon vaccination of pigs with a DNA construct expressing solely M2e and NP epitopes [185]. Taken together, these considerations suggest that the mechanism behind the VAERD phenomenon observed in pigs remains to be further studied. Also, given that no similar enhancement of disease has been observed in any other species, the swine model appears to be a unique case in this respect.

### 3.4. Nonhuman Primates

Considering the high genetic, physiologic, and anatomic similarities that non-human primates share with humans, they are considered to be a good model of the human responses to influenza infection and vaccination. This is thus a valuable tool to study human immunology, but due to ethical, economical, and practical considerations, non-human primates are not a standard for influenza virus vaccine research. Their use is particularly relevant in the case of pandemic strain vaccinations, for which challenge experiments cannot be performed in humans. For these viral strains, it is known that the cytokine responses mediate the vast majority of the pathology observed. In this respect, several studies employed functional genomics to validate the similarity of the cytokine expression through functional genomic [186] and mRNA quantification [85]. Several broadly protective vaccination approaches have been validated in this model. For example the prime/boost scheme described by Wei and colleagues [126] induced stalk mediated responses with cross H1N1 neutralizing activity. It is of note though that the neutralizing titers elicited by the different vaccination schemes tended to be lower than the levels induced in both mice and ferrets, respectively. However, this could be due to the inefficiency of DNA vaccination in large animals. Another approach for which both efficacy and breadth of protection were assessed in nonhuman primates involves immune-stimulating complex-based H3N2 vaccines [86,187], with modest results. The model is also feasible for testing of cold-adapted live attenuated vector vaccines [188].

## 4. Conclusions

In conclusion, animal models are critical for efficacy assessment and pre-clinical evaluation of novel influenza virus vaccine constructs. Several animal species have been developed and optimized for this purpose, each presenting a different set of advantages and limitations. Depending on the stage in the vaccine development process, these aspects of each model need to be considered when deciding which model is optimal for each particular antigen testing.
